# Increased interleukin-17 in the cerebrospinal fluid in sporadic Creutzfeldt-Jakob disease: a case-control study of rapidly progressive dementia

**DOI:** 10.1186/1742-2094-10-135

**Published:** 2013-11-13

**Authors:** Koji Fujita, Naoko Matsui, Yukitoshi Takahashi, Yasushi Iwasaki, Mari Yoshida, Tatsuhiko Yuasa, Yuishin Izumi, Ryuji Kaji

**Affiliations:** 1 Department of Clinical Neuroscience, The University of Tokushima Graduate School, Tokushima 770-8503, Japan; 2 National Epilepsy Center, Shizuoka Institute of Epilepsy and Neurological Disorders, Shizuoka 420-8688, Japan; 3 Department of Neuropathology, Institute for Medical Science of Aging, Aichi Medical University, Nagakute 480-1195, Japan; 4 Department of Neurology, Oyamada Memorial Spa Hospital, Yokkaichi 512-1111, Japan; 5 Department of Neurology, Kamagaya-Chiba Medical Center for Intractable Neurological Disease, Kamagaya General Hospital, Kamagaya 273-0121, Japan

**Keywords:** Creutzfeldt-Jakob disease, Cytokines, Inflammation, Interleukin-17, Neurodegeneration

## Abstract

**Background:**

Inflammatory responses in the cerebrospinal fluid (CSF) of patients with sporadic Creutzfeldt-Jakob disease (sCJD) remain elusive.

**Methods:**

We conducted a case-control study, in which 14 patients with sCJD, 14 with noninflammatory neurological disorders, and 14 with autoimmune encephalitis were enrolled. We used the suspension array system to measure the concentrations of 27 cytokines in CSF. The cytokine titers of the three groups were compared, and the correlation between the relevant cytokine titers and clinical parameters was investigated in the patients with sCJD.

**Results:**

Levels of the two cytokines interleukin (IL)-1 receptor antagonist and IL-17 were significantly elevated in the patients with sCJD compared with those in the patients with noninflammatory neurological disorders: IL-17 levels in sCJD were approximately ten times higher than in the noninflammatory neurological disorders (mean, 35.46 vs. 3.45 pg/ml; *P* < 0.001) but comparable to that in encephalitis (mean, 32.16 pg/ml). In contrast, levels of classical proinflammatory cytokines such as IL-12(p70) and tumor necrosis factor-α were increased only in encephalitis. Although not significant, IL-17 titers tended to be higher in the patients with shorter disease duration before CSF sampling (*r* = -0.452; *P* = 0.104) and in those with lower CSF total protein concentrations (*r* = -0.473; *P* = 0.086).

**Conclusions:**

IL-17 is significantly increased in CSF in sCJD, which can be an early event in the pathogenesis of sCJD.

## Background

Creutzfeldt-Jakob disease (CJD) is a rapidly progressive neurodegenerative disorder belonging to the human prion diseases and is characterized by the accumulation of prion or PrP^Sc^[1]. Immunological processes have been poorly recognized in the central nervous system (CNS) of patients with CJD, partly because pleocytosis or increased protein levels are rarely observed in the cerebrospinal fluid (CSF) of patients with CJD. However, we have detected antibodies to N-methyl-D-aspartate receptors, typically present in autoimmune limbic encephalitis [2,3], in CSF of patients with CJD, which suggests inflammatory or autoimmune responses in the CNS of patients with CJD.

Cytokines are pivotal factors in CNS inflammation in autoimmune and neurodegenerative diseases [4]. The pathogenic activities of cytokines have been vigorously investigated in prion-infected animal models [5-7]. However, there is still uncertainty regarding cytokine abnormalities, particularly in patients with CJD, because some reports suggest an increase in levels of the proinflammatory cytokines [8,9] while others suggest an increase in levels of the anti-inflammatory cytokines [10,11] in CSF. Furthermore, levels of key cytokines in autoimmune and inflammatory neurological disorders, including interleukin (IL)-17 [12], have not been evaluated in patients with CJD. We systematically measured levels of multiple proinflammatory and anti-inflammatory cytokines in CSF of patients with CJD to detect CNS inflammatory responses that can be associated with the pathogenesis of CJD.

## Methods

### Clinical and neuropathological examination

A case-control study was conducted, and patients admitted to our hospitals from April 2004 to September 2012, who were diagnosed with sporadic CJD (sCJD) according to World Health Organization criteria, were recruited [13]. The prion protein gene (*PRNP*) was analyzed in the open reading frame after extracting DNA from the patients’ blood samples [14,15]. Assays were performed to assess the CSF γ-isoform of the 14-3-3 protein (cut-off value, 500 μg/ml) [16] and total tau protein (cut-off value, 1,300 pg/ml) [17]. With regard to post-mortem examination, sections from formalin-fixed paraffin-embedded blocks of the brain were investigated. Mouse monoclonal antibody 3F4 (Dako, Glostrup, Denmark) and the EnVision amplified method (EnVision Plus kit; Dako) after hydrolytic autoclaving of the sections were used to perform immunohistochemical analysis for PrP [14,18]. Purification and Western blotting of protease-resistant PrP (PrP^Sc^) from frozen cerebral cortical samples were performed as described in a previous study [19]. Typing of PrP^Sc^ (type 1 or type 2) was performed according to the reported classification system [20].

We enrolled age-matched (≥50 years) patients with noninflammatory neurological disorders as controls and age-matched patients with noninfectious or autoimmune encephalitis in whom polymerase chain reaction was negative for herpes simplex virus-1 and virus-2, varicella-zoster virus, cytomegalovirus, Epstein-Barr virus and human herpes virus-6; autoantibodies were not necessarily screened. This study was approved by the ethics committee of the Tokushima University Hospital and was performed in accordance with the ethical standards described in the 1964 Declaration of Helsinki. Written informed consent was obtained from all participants (or guardians of participants) in the study. We obtained CSF samples by lumbar puncture at the time of evaluation from patients who were undergoing CSF examination as part of their diagnostic procedure; initial pressure, cell count, total protein level, and glucose level were measured. Remaining samples were frozen at -80°C until further investigation.

### Cytokine assay

The Bio-Plex Pro Human Cytokine 27-plex assay (M50-0KCAF0Y; Bio-Rad, Hercules, CA) was used according to the manufacturer’s instructions to evaluate the concentrations of 27 CSF cytokines and chemokines. The 27 cytokines of the panel were IL-1β; IL-1 receptor antagonist (IL-1ra); IL-2; IL-4; IL-5; IL-6; IL-7; IL-8/CXCL8; IL-9; IL-10; IL-12(p70); IL-13; IL-15; IL-17; eotaxin/CCL1; fibroblast growth factor-2 (FGF-2); granulocyte colony-stimulating factor (G-CSF); granulocyte macrophage colony-stimulating factor (GM-CSF); interferon-γ (IFN-γ); interferon-inducible protein-10 (IP-10)/CXCL10; monocyte chemotactic protein-1 (MCP-1)/CCL2; macrophage inflammatory protein (MIP)-1α/CCL3; MIP-1β/CCL4; platelet-derived growth factor BB (PDGF-BB); regulated on activation, normal T cell expressed and secreted (RANTES)/CCL5; tumor necrosis factor-α (TNF-α), and vascular endothelial growth factor (VEGF).

### Statistical analysis

The Kruskal-Wallis test was used to compare the study groups, followed by Dunn’s multiple comparison post-hoc analysis. In the multiple comparison analysis, *P* values <0.001 were considered to indicate statistical significance, and *P* values <0.05 were considered to indicate trends. Spearman’s rank correlation coefficient was then used to assess the correlations between the cytokine titers and the duration before CSF sampling, the total protein concentrations, and the total tau protein titers. In the correlation analysis, *P* values <0.05 were considered to indicate statistical significance. GraphPad Prism 5 (GraphPad Software, Inc., La Jolla, CA, USA) was used to analyze all the data.

## Results

### Clinical profiles

We enrolled 14 patients with sCJD (men, 5; age, 73.5 ± 4.9 years (mean ± standard deviation (SD))): seven with definite and seven with probable sCJD. *PRNP* was homozygous for methionine at codon 129 in all the patients. In addition, PrP^Sc^ was type 1 in five patients and type 2 in one patient. Moreover, the CSF cell count was normal in all the patients. Furthermore, the CSF 14-3-3 protein was positive in 12 of 14 patients, and the level of total tau protein was elevated in 10 of 13 patients (Table 1). The control subjects were 14 patients with noninflammatory neurological disorders (men, 10; age, 71.8 ± 10.9 years); eight had idiopathic normal pressure hydrocephalus, three had parkinsonism, two had myalgia/neuralgia and one had constant headaches. In addition, we enrolled 14 age-matched patients with noninfectious or autoimmune encephalitis (men, 8; age, 64.2 ± 7.5 years), including one with gastric cancer, one with colon cancer, one with prostate cancer, one with acoustic tumor, one with Hashimoto’s thyroiditis, one with CNS lupus and schizophrenia, one with anti-voltage-gated potassium channel complex antibodies-associated limbic encephalitis, and seven without tumors or specific autoantibodies.

**Table 1:** Clinical profiles of patients with sporadic Creutzfeldt–Jakob disease

**Number**	**Age, sex**	**Diagnosis**	**Codon 129,**	**Cell count (/μl),**	**14-3-3**	**Tau**	**Duration before sampling (months)**
			**PrP**^**Sc**^**type**	**protein (mg/dl)**	**(μg/ml)**	**(pg/ml)**	
**1**	69, M	Probable	MM	3, 27	+	Not performed	1
**2**	74, F	Definite	MM	2, 27	+	1275	4
**3**	76, M	Probable	MM	6, 60	7313	>2400	2.5
**4**	70, F	Probable	MM	5, 43	+	8860	3
**5**	76, F	Probable	MM	1, 49	23503	17340	3
**6**	74, F	Probable	MM	4, 47	8688	22750	2
**7**	75, M	Probable	MM	2, 25	0	0	1
**8**	83, F	Definite	MM1	1, 27	2244	10290	6
**9**	65, F	Probable	MM	0, 25	1256	3620	8
**10**	80, M	Definite	MM1	1, 87	15226	8520	22
**11**	74, M	Definite	MM1	1, 30	697	1707	28
**12**	74, F	Definite	MM1	2, 27	531	1930	10
**13**	73, F	Definite	MM1	1, 35	7188	>13000	4
**14**	66, F	Definite	MM2-C	1, 64	0	1180	13

C, cortical type; MM, homozygous for methionine.

### CSF cytokine profiles

**Figure 1:**
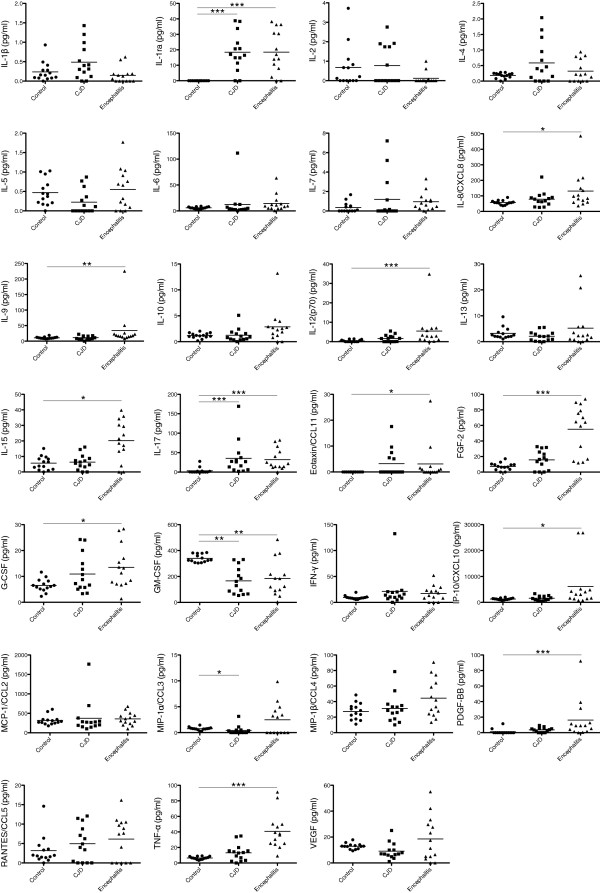
**Cytokines titers of the multiplex assay.** Cytokine titers of the control subjects (*n* = 14), the patients with sporadic Creutzfeldt-Jakob disease (CJD, *n* = 14) and those with autoimmune encephalitis (*n* = 14). *P < 0.05, **P < 0.01, ***P < 0.001.

Levels of IL-1ra and IL-17 were significantly higher in the patients with sCJD than in the controls; the IL-17 titers in the patients with sCJD were comparable to those in the patients with encephalitis and approximately ten times higher than those in the control subjects. The remaining cytokine levels were not significantly elevated in sCJD, including the classical proinflammatory cytokines IL-1β and TNF-α and the anti-inflammatory cytokines IL-4 and IL-10. The elevation of IL-12(p70), FGF-2, PDGF-BB, and TNF-α levels in the patients with encephalitis indicated clear contrast with the normal findings in those with sCJD (Table 2 and Figure 1).

**Table 2:** Cytokine profiles in the cerebrospinal fluid

**Cytokine (pg/ml)**	**Control**		**CJD**		**Encephalitis**		**P values (vs. Control)**	
	**Mean**	**SD**	**Mean**	**SD**	**Mean**	**SD**	**CJD**	**Encephalitis**
**IL-1β**	0.2371	0.2433	0.4871	0.4529	0.1471	0.2015	NS	NS
**IL-1ra**	0	0	18.43	12.38	18.49	13.81	<0.001	<0.001
**IL-2**	0.6736	1.068	0.7757	1.007	0.1200	0.3007	NS	NS
**IL-4**	0.1800	0.0854	0.5843	0.6772	0.3207	0.3526	NS	NS
**IL-5**	0.4686	0.3360	0.2250	0.3128	0.5514	0.5287	NS	NS
**IL-6**	5.911	1.834	12.36	28.79	14.48	16.42	NS	NS
**IL-7**	0.3593	0.5244	1.196	2.301	0.9507	0.9143	NS	NS
**IL-8/CXCL8**	57.38	14.30	78.63	49.37	130.9	115.9	NS	<0.05
**IL-9**	10.80	2.974	11.06	6.014	34.32	55.89	NS	<0.01
**IL-10**	1.178	0.5076	1.246	1.300	2.871	3.246	NS	NS
**IL-12(p70)**	0.4243	0.5698	1.656	1.790	5.481	8.797	NS	<0.001
**IL-13**	3.204	2.282	2.081	1.845	5.210	7.876	NS	NS
**IL-15**	5.691	4.372	6.366	4.853	20.19	13.01	NS	<0.05
**IL-17**	3.454	7.814	35.46	44.40	32.16	27.14	<0.001	<0.001
**Eotaxin/CCL11**	0	0	3.236	5.317	3.117	7.441	NS	<0.05
**FGF-2**	7.244	4.743	15.72	12.14	55.16	31.04	NS	<0.001
**G-CSF**	6.539	2.477	10.92	7.283	13.51	8.212	NS	<0.05
**GM-CSF**	339.6	30.78	166.2	103.9	184.7	139.6	<0.01	<0.01
**IFN-γ**	9.297	3.523	21.33	32.72	17.13	14.67	NS	NS
**IP-10/CXCL10**	1299	342.5	1602	866.9	6140	8906	NS	<0.05
**MCP-1/CCL2**	318.2	118.9	372.7	425.8	357.8	164.2	NS	NS
**MIP-1α/CCL3**	0.7614	0.3028	0.4243	0.8461	2.481	3.056	<0.05	NS
**MIP-1β/CCL4**	27.38	10.65	31.19	17.70	44.48	24.73	NS	NS
**PDGF-BB**	1.196	3.278	3.638	3.016	16.24	24.69	NS	<0.001
**RANTES/CCL5**	3.168	3.691	4.958	4.583	6.134	5.396	NS	NS
**TNF-α**	6.481	1.837	13.17	10.79	40.68	23.12	NS	<0.001
**VEGF**	12.88	2.234	9.086	6.164	18.51	17.16	NS	NS

CJD, Creutzfeldt-Jakob disease; G-CSF, granulocyte colony-stimulating factor; GM-CSF, granulocyte macrophage colony-stimulating factor; IFN-γ, interferon-γ; IL, interleukin; IL-1ra, IL-1 receptor antagonist; IP-10, interferon-inducible protein-10; MCP-1, monocyte chemotactic protein-1; MIP, macrophage inflammatory protein; NS, not significant; PDGF-BB, platelet-derived growth factor BB; RANTES, regulated on activation, normal T cell expressed and secreted; SD, standard deviation; TNF-α, tumor necrosis factor-α; VEGF, vascular endothelial growth factor.

### Correlation between interleukin-17 titers and clinical profiles of Creutzfeldt-Jakob disease

**Figure 2:**
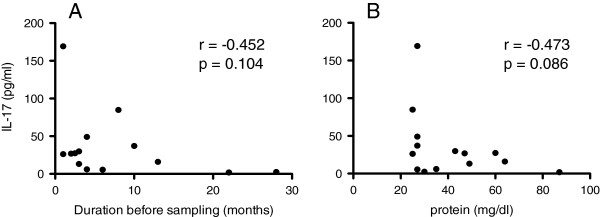
**Correlation of cytokine titers and clinical parameters. (A)** Correlation between cerebrospinal fluid interleukin-17 (IL-17) titers and disease duration before sampling in the patients with sporadic Creutzfeldt-Jakob disease. **(B)** Correlation between cerebrospinal fluid IL-17 and total protein titers in the patients with sporadic Creutzfeldt-Jakob disease. Total protein titer was used as a general inflammatory marker in the central nervous system.

We assessed the relationships between IL-17 titers and the clinical data of sCJD to investigate how elevated IL-17 levels were associated with disease progression. The IL-17 titers tended to be higher in the patients with shorter disease duration before CSF sampling (Spearman’s *r* = -0.452; *P* = 0.104), which suggested that levels of IL-17 are increased in the early phases of the disease when neuronal damage is less severe (Figure 2A). Although not significant, the IL-17 titers tended to be higher when the CSF total protein concentrations were lower (Spearman’s *r* = -0.473; *P* = 0.086; Figure 2B). There was no significant correlation between IL-17 titers and total tau protein titers (Spearman’s *r* = -0.236; *P* = 0.485), elevation of which may reflect the rate of neurodegeneration.

## Discussion

In CSF of the patients with sCJD, we detected increases in IL-17, a cytokine that induces inflammation and can be pathogenic for autoimmune neurological disorders, such as multiple sclerosis and experimental autoimmune encephalomyelitis (EAE) [12,21,22]. It is important to note that IL-17 is associated with enhanced neuroinflammation in the absence of PrP^C^, because PrP^C^ downregulation can contribute to the pathogenesis of prion disease. PrP^C^-knockout exacerbates and prolongs neuroinflammation in EAE and is accompanied by an increase in IFN-γ and IL-17 mRNA in CNS [23]. Moreover, PrP^C^-knockout can lead to enhanced EAE severity but with reduction of IL-17-positive Th17 cells [24]. These findings suggest that PrP^C^ downregulation enhances neuroinflammation and IL-17 production, but that IL-17-producing cells differ from Th17 cells. Besides Th17, IL-17 can be produced by glial cells in CNS [21], and astrocytes are critical for the IL-17 pathways in EAE [25,26]. Furthermore, IL-17 can be expressed by microglia in response to IL-1β or IL-23 [27]. Considering the rarity of T cells in CNS of patients with CJD, astrocytes and microglia can be major sources of IL-17 in CJD.

Increased IL-17 production is a new finding in prion disease, whereas the activities of other cytokines have been investigated in prion-infected mice and to a lesser extent in patients with CJD. Cytokines that are overexpressed in prion-infected mouse brain include TNF-α, IL-1α, IL-1β, IL-6, IL-12(p40), transforming growth factor-β1, MCP-1/CCL2, RANTES/CCL5, CXCL1, IP-10/CXCL10 and CXCL13 [5-7]. However, CSF findings in patients with CJD remain controversial; early studies reported an increase in levels of proinflammatory cytokines TNF-α and IL-1β [8,9], but recent studies demonstrated normal levels of these classical proinflammatory cytokines and increased levels of the anti-inflammatory cytokines IL-4 and IL-10 [10,11]. In addition, these studies reported elevated levels of IL-8/CXCL8 [11], a chemokine that can be associated with the production of IL-17 in opticospinal multiple sclerosis [28]. Of these, IL-1 can be pathogenic for prion disease because IL-1 receptor knockout mice demonstrate significantly longer prion-incubation times than controls [6]. In contrast, IL-10 can be neuroprotective because IL-10 knockout mice demonstrate significantly shorter incubation times [6,29]. Conversely, TNF-α, IL-4, IL-6, IL-12(p40), IL-12(p35), IL-13, and transforming growth factor-β1 are probably not pathogenic in prion disease because knockout of these cytokines did not change the disease course of prion-inoculated mice [5,6,29-31].

Our results were partly consistent with those of recent reports on human CSF cytokines quantified using enzyme-linked immunosorbent assay kits that revealed low titers of IL-1β, IL-6, IL-12, and TNF-α in patients with CJD [10,11]. However, the significant elevations of IL-4, IL-10, and IL-8/CXCL8 levels in CJD reported in these studies [10,11] were not observed in this study. This inconsistency may result from the different methodologies and disease controls and should be further investigated in future.

Our results imply that elevated levels of IL-17, a proinflammatory cytokine, can be an early event in sCJD rather than a mere consequence of neurodegeneration later in the disease course. This observation is intriguing because the functions of inflammation in the early stages of pathology have been vigorously investigated in several neurodegenerative diseases, such as Alzheimer’s disease, Parkinson’s disease and amyotrophic lateral sclerosis [32]. Moreover, it is worth noting that inflammatory responses may have dual functions in neurodegeneration, both as instigators of damage and as guardians of brain homeostasis. Therefore, investigation of the potential activities of IL-17 and other inflammatory markers [33,34] is warranted in prion disease.

## Abbreviations

CJD: Creutzfeldt-Jakob disease; CNS: central nervous system; CSF: cerebrospinal fluid; EAE: experimental autoimmune encephalomyelitis; FGF-2: fibroblast growth factor-2; G-CSF: granulocyte colony-stimulating factor; GM-CSF: granulocyte macrophage colony-stimulating factor; IFN-γ: interferon-γ; IL: interleukin; IL-1ra: IL-1 receptor antagonist; IP-10: interferon-inducible protein-10; MCP-1: monocyte chemotactic protein-1; MIP: macrophage inflammatory protein; PDGF-BB: platelet-derived growth factor BB; PRNP: prion protein gene; RANTES: regulated on activation, normal T cell expressed and secreted; sCJD: sporadic Creutzfeldt-Jakob disease; SD: standard deviation; TNF-α: tumor necrosis factor-α; VEGF: vascular endothelial growth factor.

## Competing interests

The authors declare that they have no competing interests.

## Authors’ contributions

KF participated in the design of the study, acquisition and analysis of data, statistical analysis and manuscript drafting and revision. NM, TY, and RK participated in data analysis and manuscript revision. YT, Y Iwasaki, and MY participated in data acquisition and manuscript revision. Y Izumi participated in acquisition and analysis of data and manuscript revision. All the authors read and approved the final manuscript.
